# Targeting Crosstalk between Nrf-2, NF-κB and Androgen Receptor Signaling in Prostate Cancer

**DOI:** 10.3390/cancers10100352

**Published:** 2018-09-25

**Authors:** Namrata Khurana, Suresh C. Sikka

**Affiliations:** 1 Department of Internal Medicine-Medical Oncology, Washington University in St. Louis Medical Campus, 660 S Euclid Ave, St. Louis, MO 63110-1010, USA; nkhurana@wustl.edu; 2 Department of Urology, Tulane University School of Medicine,1430 Tulane Avenue, New Orleans, LA 70112, USA

**Keywords:** prostate cancer, castration resistant prostate cancer (CRPC), oxidative stress, inflammation, androgen receptor (AR), AR-V7, Nrf-2, NF-κB, sulforaphane, curcumin and bardoxolone methyl

## Abstract

Oxidative stress, inflammation and androgen receptor (AR) signaling play a pivotal role in the initiation, development and progression of prostate cancer (PCa). Numerous papers in the literature have documented the interconnection between oxidative stress and inflammation; and how antioxidants can combat the inflammation. It has been shown in the literature that both oxidative stress and inflammation regulate AR, the key receptor involved in the transition of PCa to castration resistant prostate cancer (CRPC). In this review, we discuss about the importance of targeting Nrf-2-antioxidant signaling, NF-κB inflammatory response and AR signaling in PCa. Finally, we discuss about the crosstalk between these three critical pathways as well as how the anti-inflammatory antioxidant phytochemicals like sulforaphane (SFN) and curcumin (CUR), which can also target AR, can be ideal candidates in the chemoprevention of PCa.

## 1. Oxidative Stress and Prostate Cancer

Prostate cancer (PCa) is the second major cause of cancer in men and fifth leading cause of cancer-associated deaths in men worldwide [1]. Numerous studies have underscored the connection between oxidative stress and risk of developing prostate cancer [2,3]. Oxidative stress is characterized by an imbalance between the production of reactive oxygen species (ROS) and the capability of biological system to counterbalance the effects of reactive free radicals or restore oxidative damage [4]. High levels of ROS can also cause significant reduction in the antioxidant defense mechanisms resulting in DNA, protein and lipid damage [5]. The oxidative damage may be aggravated by the reduced efficacy of antioxidant defense mechanisms [6]. Hydroxyl radicals, peroxides and superoxides are ROS that can be either generated through mitochondria or extra mitochondrial NAD(P)H oxidase (Nox) system [7,8]. Oxidative stress has been shown to contribute to the initiation and progression of PCa by regulating transcription factors, cell cycle regulators and DNA [9,10]. Chronic increase in the levels of ROS causes somatic mutations as well as neoplastic transformations [11]. Antioxidant therapy may prevent PCa by combating oxidative stress [12]. Intrinsic oxidative stress can have an effect on various functions in cancer cells such as cellular proliferation, genetic instability and advancement of mutations, changes in sensitivity of the cells to anticancer agents, invasion and metastasis [13,14]. Optimal levels of intracellular ROS are critical in maintaining cellular signaling and homeostatic redox balance. Most importantly, the real effects of oxidative stress are contingent upon the genetics of the cell, the kind of ROS involved and also the duration and levels of ROS [15,16]. Oxidative stress is inherent in PCa cells and is vital for aggressive phenotype [5]. Numerous factors linked with PCa like aging, antioxidant system, androgen imbalance, dietary fat and premalignant conditions may be associated with oxidative stress [17].

**Figure 1:**
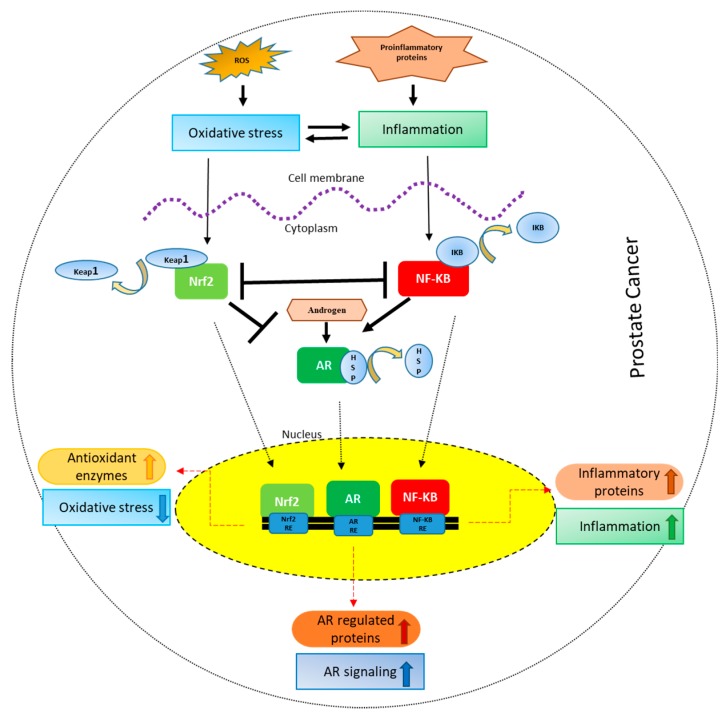
Crosstalk between Nrf-2, NF-κB and AR in PCa: Oxidative stress leads to inflammation and vice versa. Oxidative stress results in the activation and nuclear localization of Nrf-2 after its dissociation from Keap1. Nrf-2 after localizing to the nucleus binds to the antioxidant response elements leading to the transcription and translation of antioxidant proteins. Inflammation, on the other hand, leads to the activation and nuclear localization of NF-κB after its dissociation from IκB, where it binds to NF-κB response elements leading to the transcription and translation of inflammatory proteins. Nrf2 signaling inhibits NF-κB signaling and vice versa. On the other hand, Nrf-2 inhibits AR whereas NF-κB activates AR signaling. AR after binding to androgens dissociates from HSPs (heat shock proteins), translocates to the nucleus leading to the transcription and translation of AR regulated proteins like prostate specific antigen (PSA).

In response to oxidative stress, there is an activation of nuclear factor erythroid 2 -like 2 (NFE2L2; more commonly known as Nrf-2)/Kelch-like erythroid cell-derived protein with CNC homology-associated protein 1, (Keap1)signaling pathway [18]. Nrf-2 is a transcription factor regulating the basal as well as inducible expression of numerous antioxidant and detoxification enzymes [19]. Nrf-2 signaling along with other interacting and regulatory proteins and molecules is certainly the most vital defense and survival pathway employed by cells against oxidative stress [20]. Nrf-2 is normally present in the cytoplasm bound to repressor protein Keap1 which promotes its degradation by ubiquitin proteasome pathway (Figure 1).

Electrophiles (under stress conditions) alter the cysteine residues of Keap1 resulting in the prevention of ubiquitination of Nrf-2 [21]. Nrf-2 thus translocates to the nucleus where it along with the small Maf proteins binds to the antioxidant response elements present in the promoter region of target genes. This leads to the transcription and translation of antioxidant enzymes/phase II detoxifying enzymes including NAD(P)H:quinone oxidoreductase-1 (NQO-1), heme-oxygenase-1(HO-1), glutathione S-transferaseA2 (GSTA2), γ-glutamylcysteine synthetase (GCS), epoxide hydrolase, leukotriene B_4_ dehydrogenase, aflatoxin B_1_ dehydrogenase, ferritin and UDP-glucuronosyltransferase (UGT) 1A1 [22,23,24] (Table 1). Disturbance in Nrf-2 signaling are likely to enhance predisposition to oxidative damage and toxicants in humans and various model organisms [25,26,27,28,29]. Mice deficient in Nrf-2 were extremely more susceptible to carcinogens and showed augmented lung metastasis along with elevated ROS levels [30,31]. The prevalence, multiplicity and size of colorectal tumors was reported to increase in Nrf-2 knock out mice [32]. Several studies have reported the cancer chemopreventive effect of Nrf-2 activation [33,34]. During the progression of PCa in transgenic adenocarcinoma of the mouse prostate (TRAMP) mice, constant downregulation of Nrf-2 and its downstream target genes was reported [35]. Nrf-2 has been shown to suppress growth and migration of PCa cells by upregulating ferroportin [36] and sensitize PCa cells to radiation by reducing basal ROS levels [37]. Chemoprevention by phytochemicals like sulforaphane (SFN) [38,39,40,41,42] and curcumin (CUR) [43,44,45,46,47] has been attributed to the activation of Nrf-2 pathway.

## 2. Inflammation and Prostate Cancer

It has been a well-studied fact that chronic inflammation leads to the initiation and advancement of PCa by modifying the tumor microenvironment altering the balance of chemokines, cytokines, ROS and other transcriptional factors [48,49]. The extracellular matrix is remodeled which commences epithelial mesenchymal transition (EMT). The constitutively active stroma formed promotes growth of the tumor and metastatic phenotype [50]. Chronic inflammation in the specimens of benign prostate biopsy has been linked with prostate tumors of high grade in the adjoining areas [51]. In prostatic intraepithelial neoplasia (PIN) and PCa, there is a strong correlation of inflammation with proliferative inflammatory atrophy (PIA) or “risk factor” lesions comprising of activated inflammatory and proliferating epithelial cells [52].

A multitude of inflammatory cytokines have been shown to actively mediate between prostate inflammation and PCa [53]. The expression of circulating macrophage inhibitory cytokine 1 (MIC-1) was found to be augmented in PCa [54,55] and was associated with poor prognosis [56]. MIC-1 belongs to the superfamily of transforming growth factor-β (TGF-β) and has been advocated as a chief link between macrophages and PCa [57]. Interleukin 6 (IL-6) is another important cytokine whose expression has been found to increase in PCa [58]. It is secreted by macrophages, T lymphocytes and endothelial cells and is implicated in various innate and adaptive inflammatory responses such as thrombopoiesis, activation of B cells and acute-phase inflammatory response. IL-6 and IL6-receptor (IL-6R) have been also shown to be synthesized by prostate cells and their expression has been found to be upregulated in high-grade PIN and malignant epithelium [59]. The circulating levels of IL-6 were found to be augmented in castration resistant and metastatic PCa [60] with poor prognosis [61]. Interestingly, IL-6 can also play a role in activating androgen receptor (AR) [62]. Also, it was reported that a positive feedback loop between inflammation and activation of IL-6, signal transducer and activator of transcription 3 (STAT3) and nuclear factor kappa-light-chain-enhancer of activated B cells (NF-κB) retain cells in the state of epigenetic transformation [63].

NF-κB has emerged as a crucial link between inflammation and cancer [64]. The dimers of NF-κB are found to be sequestered in the cytoplasm in unstimulated cells by inhibitor of κB (IκB) through ankyrin repeat domains (Figure 1). These domains hide the nuclear localization signals (NLS) of NF-κB proteins preventing their translocation into the nucleus and thus activation [65]. NF-κB can be activated by ROS, IL-1β, tumor necrosis factor alpha (TNFα), bacterial lipopolysaccharides (LPS) and ionizing radiation. Upon activation, NF-κB translocates into the nucleus and binds to response elements on DNA leading to the transcription and translation of its target genes. Among the specific target genes of NF-κB involved in cancer, the major ones are caspase-8 inhibitor FLIP, the inhibitor of apoptosis proteins c-IAP1/2 and XIAP, B-cell lymphoma 2 (Bcl-2), Bcl-extralarge (Bcl-xL), Bax (bcl-2-like protein 4), vascular-endothelial growth factor (VEGF), basic fibroblast growth factor (bFGF), IL-8, matrix metalloproteinase-9 (MMP-9), selectins and integrins [66] (Table 1). Thus, NF-κB signaling is involved in tumorigenesis by inducing cellular proliferation, suppressing apoptosis and supporting metastasis and angiogenesis [67].

It has been reported that NF-κB mediated inflammation is even crucial in cancers whose growth is not linked with primary inflammatory condition [68,69]. In PCa, inflammation, besides being part of tumor progression, can also be elevated due to androgen deprivation therapy (ADT) mediated death of the tumor [64]. NF-κB was shown to be constitutively active in PCa [70] and the active subunit of NF-κB, p65 was found to be overexpressed in PIN as well as cancerous lesions [71]. Studies have reported that AR activity is reduced by blocking NF-κB signaling in vitro [72,73]. Another study showed that while activating NF-κB signaling did not form prostatic tumor, it did increase the rate of progression of tumor in Hi-Myc mouse PCa model [74]. It has been also shown that by blocking NF-κB signaling in PCa cells, invasion, angiogenesis and metastasis can be inhibited [75]. Interestingly, one of the studies showed that sensitivity of CRPC cells to AR antagonists can be restored by targeting NF-κB signaling using artesunate [76]. Another study showed that chemosensitivity to trichostatin A was increased in Ki-Ras-transformed human prostate epithelial cells by inhibiting NF-κB and thus this can be applied in anticancer therapy in PCa tumorigenic cells overexpressing Ki-Ras [77]. Also, NF-κB signaling activation has been shown to promote growth of PCa cells in bone [78]. The translocation of NF-κB into the nucleus was found to be upregulated in PCa lymph node metastasis [79]. Nuclear translocation of NF-κB/p65 was shown to be an independent predictive factor of biochemical relapse in PCa and also responsible for the transition from PIN to PCa [80]. Different NF-κB pathways and dimers were proposed to be activated in the progression of PCa based on the nuclear translocation of both canonical (RelA/p50) and non-canonical (RelB/p52) NF-κB subunits [81]. NF-κB was shown to activate transcription regulatory element of prostate specific antigen (PSA) gene and NF-κB binding sites were shown to be located in the PSA core enhancer [82]. The constitutive NF-κB binding activity was reported to be higher in androgen independent PCa xenografts compared to androgen-dependent PCa xenografts. These studies highlight the importance of targeting NF-κB both for the prevention (transition of healthy cells to PIN or transition from PIN to cancer) as well as the treatment for PCa.

## 3. Androgen Receptor Signaling and Prostate Cancer

Androgen receptor (AR) signaling is indispensable for the development of prostate gland [83]. It is not only responsible for the initiation of PCa but also for the progression and transition to castration resistant prostate cancer (CRPC) [84]. AR thus remains the primary therapeutic target in PCa [85]. AR consists of three major functional domains: the N-terminal domain (NTD), DNA binding domain (DBD) and C-terminal ligand binding domain (LBD). DBD is responsible for the binding of AR to androgen response elements located in the promoter region of AR dependent genes like PSA. In the absence of androgen, AR is found in the cytoplasm bound to its heat shock chaperone proteins [86] (Figure 1). When androgens bind to AR, a conformational change is induced in AR leading to dissociation of chaperone proteins and exposure of nuclear localization signal (NLS) responsible for the nuclear translocation of AR. AR then dimerizes and binds to androgen response elements resulting in the transcription and translation of the target genes. Among the specific target genes of AR involved in PCa progression, the most important ones are PSA [87], fibroblast growth factor 8 (FGF8) [88], cyclin dependent kinase 1 (Cdk1), Cdk2 [89], prostate transmembrane protein androgen induced 1 (PMEPA1) [90], transmembrane serine protease 2 (TMPRSS2) [91] and FK506 binding protein 5 (FKBP5) [92] (Table 1). Androgen deprivation therapy (ADT) using luteinizing hormone releasing hormone analogues or AR antagonists like bicalutamide, enzalutamide and flutamide so far remains the gold standard treatment for PCa patients. Although almost all patients respond to ADT initially, PCa eventually becomes resistant, leading to CRPC [93]. The major factors responsible for the development of CRPC include intratumoral/intracrine production of androgens, AR co-activators overexpression, AR gene amplification, ligand-independent activation of AR by cytokines or kinases [94,95,96] and the expression of constitutively active AR variants (AR-Vs) lacking LBD, the major one being AR-V7 [97,98].

The crosstalk between AR and other signaling pathways in PCa modulates the transactivational activity of AR. When AR function becomes dysregulated in PCa, it results in anomalous expression of AR-dependent genes including transcription factors, cell cycle regulators and proteins critical for cell survival, secretion and lipogenesis [96]. Randomized phase III studies have confirmed that AR targeting either directly or by inhibiting androgen synthesis can significantly improve the survival of metastatic CRPC patients [99]. Increased survival in PCa patients has been observed with enzalutamide [100] and abiraterone acetate [101]. Novel therapeutic approaches using agents that can directly target AR as well as siRNAs or non-coding RNAs are being developed to inhibit the growth of CRPC [102]. AR-Vs play a major role not only in the progression of CRPC and loss of sensitivity to AR targeting therapies like enzalutamide and abiraterone [103] but also in metastasis [104].

AR-V7 has been reported to be an imperative prognostic biomarker in CRPC [105,106]. AR-Vs activate AR-FL in facilitating resistance to ADT [97]. The study showed that enzalutamide could more potently prevent the growth of 22Rv1 xenograft tumors after knock down of AR-V7 highlighting the importance of targeting both AR-FL and AR-Vs for completely abrogating AR signaling. Therapeutic agents that can also target AR-Vs along with AR-FL are being currently developed to improve the therapeutic efficacy in CRPC patients [107]. We recently showed that sulforaphane (SFN) can increase the efficacy of antiandrogens like bicalutamide and enzalutamide by degrading AR in androgen dependent as well as androgen independent PCa cells [108]. We also showed that SFN can increase the efficacy of enzalutamide in enzalutamide resistant PCa cell line by degrading both AR-FL as well as AR-V7 [109].

## 4. Interplay between Nrf-2-Antioxidant, NF-κB Inflammatory and AR Signaling

Nrf-2, NF-κB and AR signaling have emerged as the most crucial signaling pathways in PCa. The interconnection between these three signaling pathways is involved in the initiation, development and progression of PCa.

### 4.1. Crosstalk between Nrf-2 and NF-κB Signaling

Nrf2 and NF-κB in addition to individually affecting several signaling pathways for maintaining a redox homeostasis also crosstalk with each other to further alter the levels of vital redox modulators in both normal and disease conditions [110]. Antitumor effect mediated by Nrf-2 is attained by both activation of antioxidant machinery as well as inhibition of NF-κB mediated pro-inflammatory pathways [111]. Oxidative stress leads to IκB kinase (IKK) activation that can cause phosphorylation of IκB, thus targeting it for polyubiquitination mediated proteasomal degradation. This results in release and nuclear translocation of NF-κB [112]. Also, oxidative stress caused due to generation of ROS by inflammatory cells is one of the key factors by which chronic inflammation leads to tumorigenesis [113]. NF-κB can directly inhibit Nrf-2 at the transcriptional level [114]. NF-κB competes with Nrf-2 for transcription co-activator CREB binding protein (CBP). Also, there is recruitment of histone deacetylase 3 (HDAC3) by NF-κB which causes local hypo acetylation hindering Nrf-2 signaling. It was reported that physical association of the N-terminal region of p65 subunit of NF-κB with Keap1 can inhibit Nrf-2 pathway [115]. Besides interacting with cytosolic Keap1, NF-κB also induced nuclear translocation of Keap1. NF-κB over-expressing cells had reduced levels of HO-1 that was stimulated by interaction of Nrf2 with antioxidant response elements confirming that activation of NF-κB can suppress transcriptional activity of Nrf-2. In endothelial cells, HO-1 prevents TNF-α mediated activation of NF-κB [116]. Inhibition of NF-κB dependent transcriptional apparatus by HO-1 has been proposed. Nuclear translocation as well as suppression of NF-κB downstream of IκB degradation could be the site of action of HO-1. This further suggests that Nrf-2 mediated upregulation of HO-1 is one of the centers for crosstalk between Nrf-2 and NF-κB.

NF-κB activation induced by LPS may be mitigated by several Nrf-2 activators such as SFN, CUR and phenethyl isothiocyanate (PEITC) [117]. Additionally, SFN and PEITC could also hinder IKK/IκB phosphorylation and nuclear translocation of p65 NF-κB subunit resulting in the inhibition of NF-κB signaling [118]. One of the studies suggested putative crosstalk between Nrf-2 and NF-κB1 regulated through mitogen-activated protein kinase (MAPK) cascade that may affect inflammation-linked etiopathogenesis of cancer [119].

Two of the most critical pathways by which SFN displays its potent chemopreventive effect in PCa are Nrf-2 activation [38,39,40,41,42] and NF-κB inhibition [38,39,118,120,121,122]. SFN induces phase II detoxification enzymes intermediated through Nrf-2 pathway thus causing elimination of electrophilic toxicants before they can cause any damage to the cellular machinery [123]. SFN interacts with Keap1, the inhibitory protein for Nrf-2, through its thiol groups [124] to modify multiple domains of Keap1 [125]. SFN can also indirectly lead to the change in conformation of Keap1 by modifying the redox status of the cell leading to the separation of Nrf-2 from Keap1 [41]. The other mechanisms by which SFN shows its effect on Nrf-2 include activation of phosphatidylinositol 3-kinase (PI3K), protein kinase C (PKC) and MAPK pathways and other epigenetic modifications which lead to phosphorylation of Nrf-2, its nuclear accumulation and finally its increased transcription and stability [40,126,127,128]. SFN augments Nrf-2 expression in TRAMP C1 PCa cells via epigenetic regulation [40]. The reduction in the growth of tumor in PCa was reported to be linked with Nrf-2 signaling activation i.e., stimulation of Nrf-2 and HO-1 proteins as well as inhibition of Keap1 protein [42].

SFN inhibits the translocation of NF-κB into the nucleus and stimulation of the expression of proinflammatory genes in PCa [129]. SFN inhibits expression of NF-κB as well as NF-κB regulated genes by inhibiting phosphorylation of IKK (especially IKKβ) and IκBα. It also reduces nuclear translocation of p65 in PC-3 PCa cells [118]. Moreover, the anti-inflammatory activity of SFN was abolished in Nrf-2 knock out macrophages signifying that antioxidant activity of SFN is related with its anti-inflammatory action [130]. In another study, SFN suppresses TNF-α stimulated NF-κB activation via inhibiting phosphorylation and degradation of IkB α and nuclear translocation of p65. The suppression correlated with the inhibition of NF-κB dependent genes linked with anti-apoptosis, cell proliferation and metastasis [120]. SFN downregulated the expression of TNF-α, inducible nitric oxide synthase (iNOS) and cyclooxygenase (Cox)-2 induced by LPS in raw macrophages [122]. The mechanism primarily responsible for SFN action was found to be suppression of NF-κB DNA binding and transactivation of NF-κB regulated genes apparently by modulating intracellular redox conditions. Similarly, anti-inflammatory effects of CUR and phenethyl isothiocyanate (PEITC) were reported to reduce with Nrf-2 knockout [45].

### 4.2. Crosstalk between NF-κB and AR Signaling

Crosstalk between androgen and pro-inflammatory signaling has been shown to remodel AR and NF-κB cistrome that reprograms transcriptome of PCa cell in a way that leads to the progression of PCa [131]. There is close proximity as well as overlap between binding sites for NF-κB and androgen receptor response elements suggesting that cooperative NF-κB and AR binding can lead to transcriptional regulation of PSA [82]. NF-κB thus can activate the expression of PSA and is found to be elevated in androgen independent PCa compared to androgen dependent PCa.

NF-κB significantly upregulates the mRNA as well as protein levels of AR, transactivation of AR, cell proliferation as well as PSA levels in the serum by binding directly to the AR promoter and activating it [73]. This was corroborated by using NF-KB inhibitors which downregulated all the above parameters in vitro. Moreover, NF-κB inhibitors also showed anti-cancer activity in androgen deprivation resistant PCa xenografts. A strong correlation was observed in the expression of AR and NF-κB in human PCa suggesting that NF-κB inhibitors can have a therapeutic potential in PCa as they can regulate the expression of AR. The compound, 3,3-diindolylmethane (DIM) was shown to inhibit cellular proliferation and induce apoptosis in androgen dependent LNCaP and androgen independent C4-2B PCa cell lines by disrupting the potential crosstalk between Akt, NF-κB and AR through significant inhibition of NF-κB DNA binding activity, Akt activation as well as AR phosphorylation and the expression of AR and PSA [132]. DIM further suppressed nuclear translocation of AR resulting in the downregulation of AR target genes. AR activation as well as elevated expression of PSA by IL-4 was repressed by IκBα. Further, IL-4 stimulated NF-κB activation and nuclear translocation was shown to be blocked by PI3K/Akt inhibitor [133]. This study showed that crosstalk between IL-4, Akt and NF-κB signaling pathways can play a vital role in the transition of androgen dependent PCa to CRPC by activating AR signaling. The resistance of CRPC cells to antiandrogens was overcome by targeting NF-κB signaling using artesunate [76]. The combination was able to reduce NF-κB signaling as well as decrease the expression of AR and AR-V7 through ubiquitin proteasomal pathway. This study highlighted that the combination of NF-κB inhibitors and AR antagonists may potentiate the clinical efficacy in CRPC patients. The enhanced PCa severity may activate NF-κB and AR-V7 [134]. The forced activation of NF-κB could induce AR-V7 expression in human prostate cells leading to resistance to 5α-reductase inhibitor (5ARI) treatment. This signified a possible mechanism by which patients can develop resistance to 5ARI. One of the studies showed that secretory proteins from neuroendocrine cells were able to stimulate NF-κB signaling in androgen dependent PCa cells that increased active AR levels [72]. Inhibition of AR activation was achieved by inhibiting NF-κB signaling in vitro. Furthermore, absence of IκBα inhibitor resulted in constant activation of NF-κB signaling in vivo which prevented regression of the prostate after castration. High nuclear levels of AR were sustained along with the maintenance of differentiated function and renewed proliferation of the epithelium. This study showed that activation of NF-κB pathway by neuroendocrine secretory proteins could sustain androgen independent growth of PCa by regulating AR. One of the other reports studying inflammation in benign prostate hyperplasia patients showed that patients having potent immune inflammation had larger volumes of prostate, increased AR and serum PSA levels [135]. ADT in mice activates an inflammatory response by the dying PCa cells causing the permeation of B and T cells [136]. The B cells produce lymphotoxin and other factors that enhance IKKα and Stat3 signaling critical for the androgen independent survival of PCa cells. These studies demonstrate the importance of targeting NF-κB signaling in PCa to abrogate AR signaling.

### 4.3. Crosstalk between Nrf-2 and AR Signaling

Oxidative stress contributes to the initiation, progression and transition of PCa to CRPC by activating AR signaling [137]. Thus reducing oxidative stress by the use of therapeutic agents that can activate Nrf-2 can prevent the conversion of PCa to CRPC. Nrf-2 has been shown to decrease transactivation of AR by inducing the nuclear accumulation of p120-Nrf1 [138]. In this study, the overexpression of Nrf-2 was shown to significantly suppress dihydrotestosterone (DHT) induced activity of AR. This AR downregulation effect of Nrf-2 overexpression was seen both under basal as well as DHT induced conditions in the androgen dependent PCa cell line (LNCaP) but only under DHT induced conditions in androgen independent PCa cell line (C4-2B). Moreover, Nrf-2 overexpression was also able to decrease nuclear levels of AR under DHT stimulated conditions in LNCaP cells but not in C4-2B cells. This study thus demonstrated a unique mechanism by which oxidative stress stimulated transcription factors are used by PCa cells to enhance AR function notwithstanding low androgen levels during ADT. The balance between Nrf-1 and Nrf-2 is very critical in regulating activity of AR as well as oxidative stress in PCa cells [139].

We recently showed that bardoxolone methyl (BM) which is a potent Nrf-2 inducer can increase the efficacy of enzalutamide in an enzalutamide-resistant PCa cell line (CWR22Rv1) by degrading both AR-FL as well as AR-V7 further underscoring the AR suppressive function of Nrf-2 [109]. This corroborates the fact that AR downregulation and Nrf-2 activation are the major pathways responsible for the anticancer efficacy of phytochemicals like SFN [38,39,40,41,42] and CUR [43,44,45,46,47] in PCa. SFN has been shown to augment expression of Nrf2 in mouse TRAMP C1 PCa cells [40] and cause transcriptional repression [140] as well as destabilization of AR in PCa cells [141]. We also showed in two of our recent studies that SFN can degrade both AR-FL and AR-V7 in PCa cell lines and thus increase the efficacy of antiandrogens in both androgen dependent as well as androgen independent PCa cell lines [108,109]. Similar to SFN, CUR was reported to reactivate Nrf-2 signaling in mouse TRAMP C1 PCa cells through epigenetic regulation [46] and downregulate gene expression and activation of AR in both androgen dependent as well as androgen independent PCa cell lines [142,143]. CUR analog Ca27 downregulates AR in PCa cells through oxidative stress mediated mechanism as well as activate Nrf-2 and Nrf-2 regulated genes [144]. Thus, activation of Nrf-2 pathway can lead to the downregulation of AR signaling in PCa.

## 5. Conclusions

Oxidative stress and inflammation are the key players involved in the initiation and progression of PCa because of their potential to modulate AR signaling. Thus the integrated signaling network involving Nrf-2, NF-κB and AR plays a very critical role both in the development and therapy of PCa (Figure 1). Therefore, there is a critical need for a therapy that can simultaneously target oxidative stress, inflammation and AR signaling in PCa. Phytochemicals, the chemical compounds produced by plants, can exert significant biological effects on human cells. They may have promising anti-cancer effect due to their ability to modulate a number of molecular signaling pathways involved in tumorigenesis [145].

**Figure 2:**
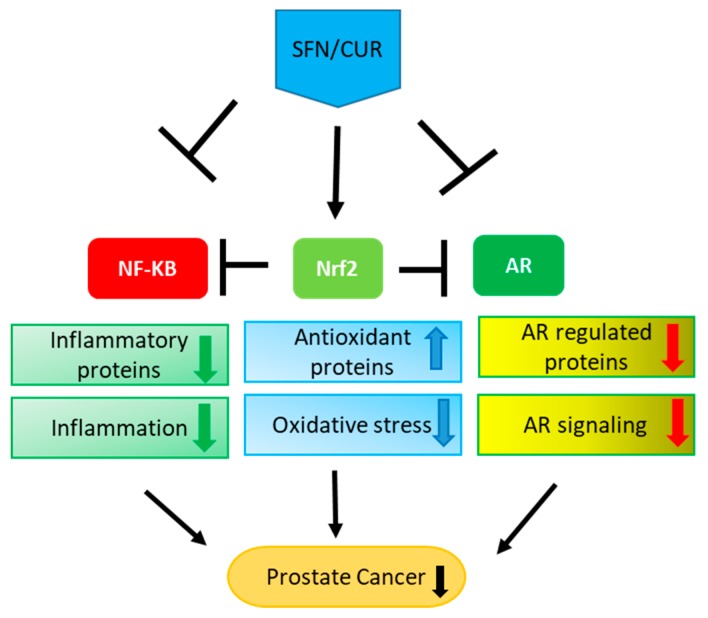
Multiple effects of SFN/CUR: SFN/CUR shows its anti-cancer effect in PCa by activation of Nrf-2 signaling and inhibition of NF-κB as well as AR signaling. As these three pathways are interlinked with each other, it leads to the upregulation of antioxidant proteins and downregulation of inflammatory proteins eventually resulting in the overall suppression of AR signaling and PCa growth.

Numerous studies have shown the anti-cancer efficacy of SFN, an isothiocyanate derived from broccoli in various different kinds of cancers [38,39,121,146,147]. Similarly, curcumin has been shown to have promising anti-cancer effects [44,46,142,143,144]. Due to their ability to concurrently target oxidative stress, inflammation and AR signaling in PCa, SFN and CUR may be very beneficial and promising in treating PCa, at the initiation as well as at the later stage (Figure 2). However, low bioavailability of phytochemicals is a major challenge. To overcome this, liposomal and other nanoformulations are being currently used to enhance their solubility in water and thus their bioavailability [148,149,150,151]. As phytochemicals are derived from natural sources, they are generally considered to be safe. However, they can show toxic effect at certain concentrations and situations like drug-drug interaction [152]. These limitations can hinder the clinical and chemopreventive applications of phytochemicals. Therefore, a comprehensive knowledge of the phytochemicals and their pharmacological effects is very critical for their transition from bench side to bedside (Figure 2).

## Figures and Tables

**Table 1:** Downstream target genes of Nrf-2, NF-κB and AR.

Downstream Target Genes	
Nrf-2	NAD(P)H:quinone oxidoreductase-1 (NQO-1), heme-oxygenase-1 (HO-1), glutathione S-transferaseA2 (GSTA2), γ-glutamylcysteine synthetase (GCS), epoxide hydrolase, leukotriene B_4_ dehydrogenase, aflatoxin B_1_ dehydrogenase, ferritin and UDP-glucuronosyltransferase (UGT) 1A1
NF-κB	Caspase-8 inhibitor FLIP, the inhibitor of apoptosis proteins c-IAP1/2 and XIAP, B-cell lymphoma 2 (Bcl-2), Bcl-extralarge (Bcl-xL), Bax (bcl-2-like protein 4), vascular-endothelial growth factor (VEGF), basic fibroblast growth factor (bFGF), IL-8, matrix metalloproteinase-9 (MMP-9), selectins and integrins
AR	Prostate specific antigen (PSA), fibroblast growth factor 8 (FGF8), cyclin dependent kinase 1 (Cdk1), Cdk2, prostate transmembrane protein androgen induced 1 (PMEPA1), transmembrane serine protease 2 (TMPRSS2) and FK506 binding protein 5 (FKBP5)
